# The impact of spectral data pre‐processing on the assessment of red wine vintage through spectroscopic methods

**DOI:** 10.1002/jsfa.14351

**Published:** 2025-05-12

**Authors:** Aristeidis S Tsagkaris, Natasa Kalogiouri, Viola Tokarova, Jana Hajslova

**Affiliations:** ^1^ Department of Food Analysis and Nutrition, Faculty of Food and Biochemical Technology University of Chemistry and Technology Prague Prague Czech Republic; ^2^ Laboratory of Analytical Chemistry, Department of Chemistry Aristotle University of Thessaloniki Thessaloniki Greece; ^3^ Department of Chemical Engineering, Faculty of Chemical Engineering University of Chemistry and Technology Prague Prague Czech Republic

**Keywords:** wine authenticity, spectral pre‐processing, chemometrics, absorption spectroscopy, attenuated total reflectance Fourier transform infrared spectroscopy

## Abstract

**Background:**

Red wine is a common target of fraudulent acts considering its high market value and popularity. Although there has been much effort to assess the geographical and varietal origin of wine, this is not the case for wine vintage. Vintage is a crucial parameter for the market price, especially in the case of reputable wines. Considering the season‐to‐season variations affecting wine quality and the ever‐occurring unstable climatological conditions due to climate change, developing analytical strategies to accurately assess wine vintage is topical and of high interest.

**Results:**

In this study, we successfully employed ultraviolet–visible spectroscopy, fluorescence spectroscopy and mid‐infrared spectroscopy to identify the vintage of a protected designation of origin red wine produced during four different vintages (*n* = 36). Class‐based clustering and great discriminatory performance was achieved for the majority of the developed multivariate models and the impact of the applied spectral pre‐processing was significant. Importantly, the tested scatter correction methods resulted in the best cross‐validation parameters (goodness of fit, R^2^Y > 0.9 and goodness of prediction, Q^2^Y > 0.8) with calculated recognition and prediction abilities in the range 77–100% and 65–96%, respectively, when using partial least squares discriminant analysis. In addition, in the case of fluorescence spectroscopy, a batch effect was revealed, which was compensated by the spectral pre‐processing methods. Spectral feature selection was performed in all cases to use only the analytically important spectral signals and omit model overfitting.

**Conclusions:**

The developed method is simple, cost‐efficient and non‐destructive, indicating its high potential for industrial applications as a rapid screening tool. © 2025 The Author(s). *Journal of the Science of Food and Agriculture* published by John Wiley & Sons Ltd on behalf of Society of Chemical Industry.

## INTRODUCTION

Wine is an alcoholic beverage, mainly produced from *Vitis vinifera* grapes, featuring a significant market price and a long winemaking tradition in Europe.^1^ According to a recent briefing in the European Parliament, the EU accounted for 48% of global wine consumption^2^ indicating high popularity of wine among the public. In this way, fraudulent practices targeting wine are common in the market including, but not limited to, dilution with water or fruit juices, mislabeling or sugar addition^3^ highlighting the need to secure wine authenticity protecting both producers and consumers. In fact, wine authenticity represents a unique case as quality schemes were already established in France in 1919 using the term ‘appellations d'origine’,^4^ followed by the ‘quality wines produced in specified regions’ scheme (European Economic Community, Regulations 816/70 and 817/70) and today's common organization of the wine market in the EU including the ‘protected designation of origin’ (PDO) scheme (Regulation 479/2008). Obviously, geographical indications have been under the spotlight resulting in a plethora of analytical methods, e.g. isotope ratio mass spectrometry, to assess wine geographical origin, especially of monovarietal wine.^5^ Nevertheless, this has not been the case for other wine authenticity concepts, such as the vintage of fine wines.^6^

The vintage is a critical parameter impacting the market price of reputable wines.^7^ This is because climatological season‐to‐season variations alter the final product composition, i.e. the content of phenolics and anthocyanins as well as volatile compounds impacting aroma.^8^ For example, in the case of Bordeaux wine, it is believed that mild winters without frost, insolation and limited precipitation before harvesting promote sugar concentration resulting in favorable flavors.^9^ In this way, much attention has been paid to correlate vintage rankings, published by internationally recognized organizations, to climate variables (temperature, precipitation, sunny days, etc.) identifying viticultural practices enhancing wine quality.^10^ However, such correlations are becoming more challenging as unprecedented climatological conditions become more and more evident, due to climate change,^11^ impacting the quality of produced wine and indicating the potential of fraudulent acts to become more frequent.^12^ In such scenarios, mislabeling (using a false vintage) of a monovarietal wine can be highly probable indicating the need to employ analytical methods capable of efficiently distinguishing among different wine vintages.

Wine vintage assessment has not attracted much attention so far and a limited number of studies have been published with chromatographic methods being the primary choice.^13^ Liquid chromatography coupled to mass spectrometry or conventional readers, such as diode array detectors, has been successfully used to discriminate vintage based on the wine phenolic content. For example, Liu *et al*. combined chromatographic analysis with chemometrics and exploited 13 different phenolic compounds, e.g. gallic acid and myricetin, to differentiate among Cabernet Sauvignon wines with different vintages.^14^ Nevertheless, chromatographic analysis requires experienced personnel and costly instrumentation limiting the opportunities for cost‐efficient analysis. This can be feasibly addressed by applying spectroscopic techniques^15^ including ultraviolet (UV)–visible spectroscopy and mid‐infrared (MIR) spectroscopy.

In this context, we investigated the potential of three spectroscopic methods to assess the vintage of the Greek PDO ‘Xinomavro Naoussa’ wine. UV–visible absorption (Abs) and fluorescence (FL) spectroscopies as well as attenuated total reflectance Fourier transform infrared (ATR‐FTIR) spectroscopy were employed. Our hypothesis was that the climatological conditions occurring during a specific vintage would have an effect on wine composition that could be identified through non‐destructive spectroscopic analysis. Considering the complexity of the spectral fingerprints, it was necessary to process them using statistical and chemometric tools. In this way, 12 different spectral pre‐processing methods were tested for each technique and their impact on the applied chemometrics performance was evaluated. Upon identifying the most promising spectral pre‐processing for each method, spectral feature selection was performed to use only the analytically important spectral signals and omit model overfitting.

## MATERIALS AND METHODS

### Wine samples

Initially, 36 (*n* = 36) monovarietal red wine samples belonging to the PDO Xinomavro variety from Naoussa (Imathia regional unit of Macedonia, Greece) produced during four different vintages (1998, 2003, 2008 and 2015) were donated by local producers. Xinomavro production is very localized, with vineyards covering a relatively small region of 700 ha indicating quite similar cultivation conditions.^16^ It is important to mention that all tested wine bottles originated from the same vineyard, following the same winemaking, aging and bottling processes. All the provided samples were kept under the same conditions of aging in a cellar and were transported to our laboratory before analysis. This ensures that the monitored differences are predominantly related to the vintage. For each vintage, nine bottles were available. Upon identification of the models with the optimum performance (see Results and Discussion), another six samples of PDO Xinomavro from 2019 were purchased from the market. For these six samples, UV–visible and FTIR analyses were performed.

In terms of the analysis, each bottle was opened immediately before each spectroscopic analysis to avoid the impact of oxidative reactions on the acquired spectra. To identify potential differences in the recorded spectral responses, the samples were split in three batches (each batch contained 12 samples, three from each vintage) and the samples were measured over a three‐month period (one batch per month). For all tested vintages, the first batch included samples with numerical ID 1–3, the second batch 4–6 and the third batch 7–9. In the case of the FL measurements, in total 30 samples were monitored as experimental error occurred during the analysis of the third sample batch (vintages 1998 and 2003).

### UV–visible spectroscopy

To record the Abs and FL fingerprints in the UV–visible spectral region, two different spectrophotometers were used. In the case of Abs, an Epoch BioTek reader (Winooski, VT, USA) was utilized and the spectra were recorded in the 300–700 nm region with a spectral resolution of 2 nm. For FL, an Infinite® 200 PRO reader (Tecan, Switzerland) was used. The excitation wavelength (*λ*_ex_) was set at 250 nm and the emission spectra were recorded in the region 280–700 nm with a spectral resolution of 2 nm, based on a recently published study on wine authenticity.^17^ In both cases, to evaluate the impact of the matrix effect on the spectral acquisition, each sample was measured (i) without dilution, (ii) two‐times dilution, (iii) five‐times dilution and (iv) ten‐times dilution. Phosphate buffer saline (PBS; Sigma Aldrich, Prague, Czech Republic) was used to dilute the wine samples. PBS tablets were used and a tablet was diluted in deionized water resulting in a 0.01 mol L^−1^ solution. Each measurement was performed in triplicate using 96‐microwell plates (Gama Group, Ceske Budejovice, Czech Republic) and the mean Abs and FL values were calculated and used during spectral pre‐processing and chemometrics.

### FTIR spectroscopy

To acquire the absorption spectra in the MIR region the settings of our recently published study^18^ were used with slight modifications. Briefly, a Nicolet iS50 FTIR (Thermo Fisher Scientific, USA) instrument was used featuring a deuterated triglycine sulfate detector and KBr as a beam splitter. All samples were analyzed using the ATR mode (diamond crystal) and the acquired absorption spectra covered the MIR region, 400–4000 cm^−1^. The number of scans for background and sample collection was set to 64, the resolution to 2 cm^−1^ and data spacing to 1.928 cm^−1^. Each sample was monitored in triplicate by covering the ATR crystal with a wine sample. The acquired results were processed utilizing OMNIC™ software (Thermo Fischer Scientific, Nicolet CZ, Prague, Czech Republic). After each sample triplicate, background collection was performed. For chemometric analysis, the acquired spectra were exported to .xlsx files using MS Excel 2019.

### Spectral pre‐processing

Identifying the most appropriate pre‐processing method for a specific food matrix, in this case red wine, is of utmost importance. This has been shown to be particularly beneficial in wine authentication studies, including vintage authentication,^19^ where the quality of spectral data directly affects the accuracy of classification models.^20^, ^21^ Optimizing these pre‐processing strategies can significantly impact the reliability of chemometric models.^22^ In this way, three different pre‐processing methods were applied, namely (i) scatter correction methods, (ii) spectral derivation methods (or noise correction methods) and (iii) their combinations, resulting in 12 different spectral data pre‐processing methods (Table 1). For a deeper understanding of the spectral pre‐processing method role in spectroscopy studies, Lee *et al*.^23^ and Hellin *et al*.^24^ are recommended. A SIMCA package (version 13.0.3.0; Göttingen, Germany, date of release: 14.03.2014) was utilized to accomplish all the pre‐processing and chemometric analysis. Data normalization was performed using Pareto scaling resulting in feature‐wise transformations to adjust the variance of the different features,^25^ especially reducing the relative importance of large values.^26^

**Table 1:** Applied spectral pre‐processing methods alongside their assigned ID number. These IDs are used in the rest tables representing a specific pre‐processing method in each case

ID	Pre‐processing method	Type of pre‐processing
1	Raw data	None
2	Multiplicative scatter correction (MSC)	Scatter correction methods
3	Standard normal variate (SNV)	
4	Savitzky–Golay (SG) 1st derivative 2nd polynomial	Spectral derivatives
5	SG 1st derivative 3rd polynomial	
6	SG 2nd derivative 3rd polynomial	
7	MSC followed by SG 1st derivative 2nd polynomial	Combined
8	MSC followed by SG 1st derivative 3rd polynomial	
9	MSC followed by SG 2nd derivative 3rd polynomial	
10	SNV followed by SG 1st derivative 2nd polynomial	
11	SNV followed by SG 1st derivative 3rd polynomial	
12	SNV followed by SG 2nd derivative 3rd polynomial	

### Applied chemometrics

After completing the spectral pre‐processing, both non‐supervised and supervised chemometrics were applied, namely principal component analysis (PCA) and partial least squares discriminant analysis (PLS‐DA), respectively, and multiclass comparison was performed. In terms of PLS‐DA, a sevenfold cross‐validation was performed and the goodness of fitting (R^2^Y) and goodness of prediction (Q^2^Y) were used to evaluate the impact of each spectral pre‐processing. For the models achieving the best cross‐validation parameters, follow‐up chemometric analysis was performed. Firstly, a variable importance for the projection (VIP) score was utilized for identifying the most significant spectral features and a cut‐off limit was set to 1.0. This ensured that the developed models are robust and are not prone to overfitting as only spectral features with analytically important information were used.^27^

Based on these results, the models performing the best (2 for Abs, 2 for FL and 1 for ATR‐FTIR) were chosen and an external model validation was applied. Considering the sample set size, each class was randomly split seven times into training set (*n* = 7) and test set (*n* = 2) (different training and test set every time) and the recognition and prediction abilities were calculated. Recognition ability was calculated as the percentage of correctly classified training set samples during the modeling step. Prediction ability was also monitored by determining the mean value of the correctly classified test samples when using the models developed in the training step.^28^ For these five models, root mean square error of estimation (RMSEE) was also reported, expressing the fit of the model.^29^ The following formula was used:

$$\sqrt{\frac{\sum {\left(Y_{\mathrm{obs}}-Y_{\text{pred}}\right)}^{2}}{N-1-A}}$$

where *Y*_obs_ − *Y*_pred_ refers to the fitted residuals for the observations in the workset, *N* refers to the number of observations and *A* refers to the number of components. Analysis of variance of the cross‐validated residuals (CV‐ANOVA) was calculated expressing the model reliability. Lastly, 100 random permutations were performed to investigate the model validity and the degree of potential overfitting. RMSEE, CV‐ANOVA and the permutation tests were performed using SIMCA 13.0.3.0.

## RESULTS AND DISCUSSION

### UV–visible spectral interpretation

#### Abs spectra

**Figure 1:**
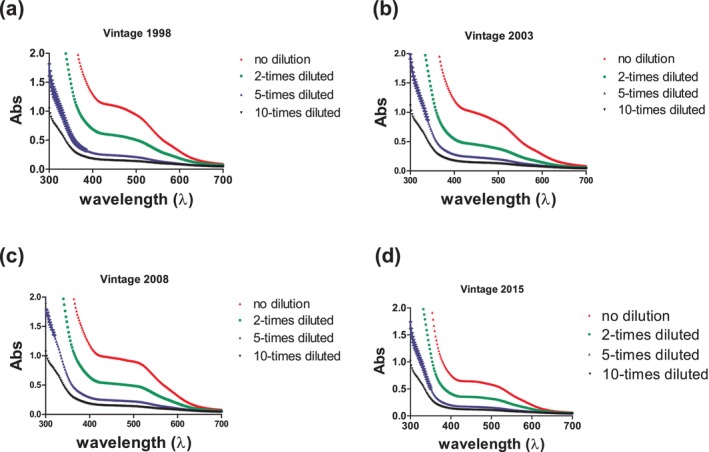
Indicative Abs spectra for (a–d) each analyzed vintage. Each color represents a different dilution as displayed in the legend.

Wines produced in four different vintages were tested and the acquired UV–visible Abs spectra are presented in Fig. 1. The monitored pattern was similar in all cases; the non‐diluted samples recorded the highest Abs and a descending trend was noticed as the dilution rate increased. The spectral shoulders around 300–320 nm might be related to galloyl (galloylated flavanols) and acetylated anthocyanins.^19^ In addition, a sharp signal decrease was recorded on diluting the samples in this region and this decrease was proportional to the dilution step. Significant Abs was observed between 400 and 550 nm, which is characteristic to red pigments such as anthocyanins. In fact, the Abs at 420 and 520 nm can be used to calculate the so‐called ‘color index’ accounting for the total anthocyanin content in wine, a parameter that can be impacted by wine age^30^ and pH.^31^

#### FL spectra

**Figure 2:**
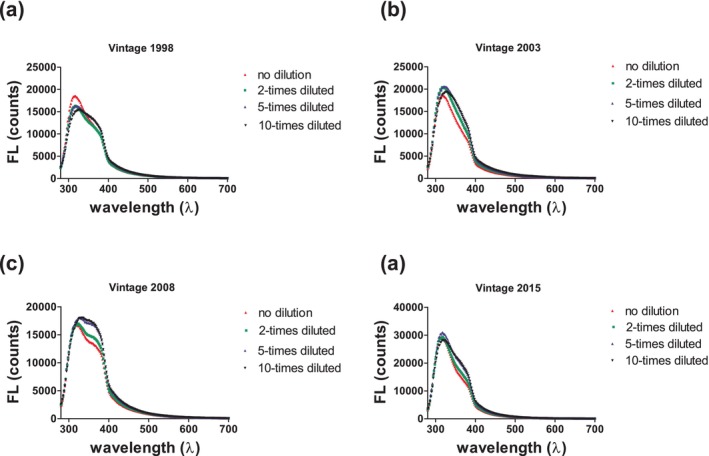
Indicative FL emission spectra for (a–d) each analyzed vintage. Each color represents a different dilution as displayed in the legend.

A strong spectral band was observed for all tested samples between 280 and 400 nm and its intensity varied for the four different vintages (Fig. 2). The highest intensity was observed for wines with vintage in 2015 whilst similar intensity was seen for the other three groups. The obtained signal can be related to several wine components acting as fluorophores, e.g. phenolic compounds, coumarins, vitamin B2 as well as compounds originating from wooden barrels and generated during wine aging.^32^ A quenching effect was also identified in the non‐diluted samples resulting in a lower signal (Fig. 2(b)–(d)). Certain wine components may act as FL quenchers,^33^ e.g. tannins, explaining why lower signal was detected for the more concentrated samples. In most cases, the five‐times diluted samples exhibited the highest signal. Similar FL emission spectra were recently recorded within a wine authenticity study investigating various wine varieties^17^ using the same *λ*_ex_ as in our study. It is important to notice that depending on the excitation wavelength, a different emission spectrum is obtained. In our study, a single excitation wavelength was selected to enhance method simplicity as well as considering that another two spectroscopic methods were used to test the wine samples. On the other hand, by using multiple excitation wavelengths (264, 290 and 330 nm), Wu *et al*.^34^ recently identified three emission spectral bands, i.e. 372, 371 and 407 nm, respectively. These bands were attributed to several phenolic acids, flavonoids and flavan‐3‐ols.

### Investigating UV–visible spectral data pre‐processing and impact on multivariate analysis

#### Sample clustering using Abs spectra

In total 12 pre‐processing methods were evaluated for a four‐class model (vintages 1998, 2003, 2008, 2015) and the obtained results are presented in Table 2. Based on the obtained cross‐validation parameters, the model performance was rather poor in the case of non‐diluted and ten‐times diluted samples. Actually, in most of these models the R^2^Y was below 0.40 indicating a poor model predictive ability. Usually, a significance threshold of 0.5 is used in food authenticity studies as a quality criterion.^35^ In the case of non‐diluted samples, the wine matrix was too concentrated resulting in spectral saturation which hindered the obtaining of analytically useful information. A 10‐time dilution factor was proven to dilute too much the tested samples and to reduce the amount of analytically useful information. On the other hand, when the samples were two‐ and five‐times diluted, the cross‐validation performance was very good (i.e. Q^2^Y > 0.5) for most of the applied pre‐processing methods. Within this context, further discussion for these chemometric models is provided below.

**Table 2:** Effect of spectral data pre‐processing methods on the obtained cross‐validation parameters in the case of Abs spectroscopy (*n* = 36)

Applied pre‐processing ID	No dilution		Two‐times dilution		Five‐times dilution		Ten‐times dilution	
	R^2^Y	Q^2^Y	R^2^Y	Q^2^Y	R^2^Y	Q^2^Y	R^2^Y	Q^2^Y
1	0.237	0.171	0.759	0.619	0.953	0.878	0.973	0.919
2	0.199	0.012	0.863	0.724	0.909	0.832	0.227	0.135
3	0.203	0.019	0.96	0.881	0.891	0.812	0.229	0.139
4	0.267	0.0955	0.965	0.846	0.916	0.757	0.261	0.146
5	0.924	0.863	0.943	0.775	0.907	0.749	0.263	0.161
6	0.129	0.0752	0.218	0.0838	0.896	0.654	0.245	0.181
7	0.238	0.172	0.965	0.854	0.916	0.742	0.264	0.156
8	0.966	0.904	0.949	0.799	0.891	0.738	0.272	0.167
9	0.0972	0.0534	0.219	0.0742	0.614	0.438	0.244	0.181
10	0.624	0.571	0.966	0.86	0.916	0.742	0.266	0.159
11	0.966	0.905	0.949	0.796	0.89	0.733	0.243	0.18
12	0.104	0.0586	0.219	0.0757	0.614	0.436	0.243	0.18

**Figure 3:**
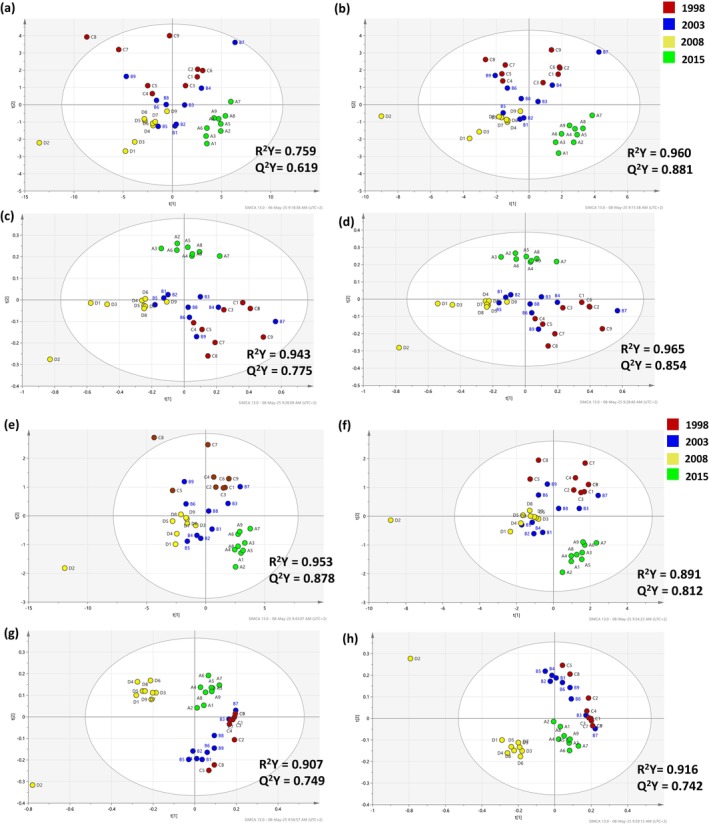
PLS‐DA score plots after applying different pre‐processing methods for the two‐times diluted (a–d) and five‐times diluted (e–h) samples. The data were obtained by monitoring Abs in the UV–visible region. The loading plots for the respective PLS‐DA models can be found in the supporting information (Fig. S3). (a) and (e): raw data; (b) and (f): SNV; (c) and (g): SG 1st derivation (3rd polynomial); (d) and (h): MSC & SG 1st derivation (2nd polynomial).

To monitor the distribution of the wine samples towards their vintage, non‐supervised PCA was applied and four cases are indicatively presented. In terms of the two‐times dilution (Fig. S1), improved class‐based clustering was achieved by using scatter correction methods such as standard normal variate (SNV; Fig. S1(b)). On the other hand, when noise correction methods, e.g. derivatives, or their combinations were applied to scatter correction methods then the class‐based clustering was not profound. Interestingly, by increasing the dilution factor to 5, a much better clustering was achieved for the noise correction methods (Fig. S1(g),(f)) as fewer matrix‐related spectral interferences were present (due to the higher dilution). This indicates that dilution is a simple and effective measure for reducing the matrix background light scattering, absorption and fluorescence emission, compensating the direct optical interference of the wine matrix.^36^ The loading plots for all PCA models can be found in the supporting informations S1 and depending the applied pre‐processing a slightly different distribution of the loadings was noticed (Fig. S2). Subsequently, supervised PLS‐DA was performed (Fig. 3) and in the case of two‐times dilution, R^2^Y and Q^2^Y fluctuated in the range 0.759–0.965 and 0.619–0.881, respectively. Important to notice is that when the third polynomial fitting was used the cross‐validation parameters were rather poor (Table 2). Slightly better results were attained with the five‐times dilution. In this case, all of the tested pre‐processing resulted in cross‐validation parameters at the range 0.61–0.953 (R^2^Y) and 0.438–0.878 (Q^2^Y) whilst no problem with the third polynomial fitting was observed. Unexpectedly, the model built using raw data (without any spectral pre‐processing) achieved the highest R^2^Y score (R^2^Y = 0.953) indicating that an appropriate dilution factor can enhance the discriminatory performance of a chemometric model.

As a final step, it is necessary to identify the spectral features resulting in sample discrimination. This was performed for the models with the highest proximity between R^2^Y and Q^2^Y (one model for the two‐times diluted and one for the five‐times diluted samples), as this ensures that the explained variance is described in the most predictable way. Table 3 summarizes the PLS‐DA models for which spectral feature selection was performed and a detailed list of the spectral features alongside their VIP scores is provided in the supporting information (excel file). In the case of the two‐times diluted samples, the SNV model exhibited the best performance with R^2^Y = 0.96 and Q^2^Y = 0.881. It was found that 80 spectral features achieved a VIP score higher than 1.0 with the majority of them originating from the UV part of the spectrum. The highest VIP score was achieved for *λ* = 296 nm (VIP_296nm_ = 2.12) representing a spectral region close to the absorption maxima of flavanol monomers and polymers.^37^ Interestingly, most of the spectral features impacting the sample clustering in the PCA loading plot (Fig. S2(b)) belong to the UV region. In terms of the five‐times diluted samples, multiplicative scatter correction (MSC) was considered the most effective spectral pre‐processing (R^2^Y = 0.909 and Q^2^Y = 0.832). In this case, 78 spectral features exceeded the cut‐off score and the highest VIP score was attained for *λ* = 290 nm (VIP_290nm_ = 3.67). These findings are in line with the literature, as Philippidis *et al*. used the spectral region around 290 nm as a marker for the discrimination of Greek red wine varieties using UV–visible spectroscopy.^38^

**Table 3:** PLS‐DA models with the highest conformity between R^2^Y and Q^2^Y for each of the tested spectroscopic methods

Spectroscopic method	Dilution	Spectral pre‐processing	Spectral features with VIP > 1.0	
			UV	Visible
Abs	Two times	SNV	54	26
	Five times	MSC	42	36
FL	No dilution	SNV	60	0
	Two times		60	2

#### Sample clustering using FL spectra

The effect of the tested spectral pre‐processing methods was different when the FL spectral fingerprint was monitored (Table 4). In detail, the scatter correction methods, namely SNV and MSC, provided the best results for the non‐diluted samples, in terms of the monitored cross‐validation parameters. Wine may contain sediments (result of fermentation or maturation) as well as tartrate crystals, compounds that may interact with the illumination radiation resulting in potential signal artefacts. Thus, by using scatter correction methods, the potential scattering effect induced by such wine compounds was compensated. On the other hand, noise correction methods were more effective when diluted samples were measured. Dilution can reduce the interfering effect of certain compounds compensating matrix effects.^39^ This in combination with the enhanced resolution and reduced background achieved by the spectral derivation resulted in R^2^Y values ranging from 0.469 to 0.723 for the two‐times diluted samples and from 0.406 to 0.726 for the five‐times diluted samples. In terms of the ten‐times diluted samples, the enhanced sensitivity provided by FL spectroscopy provided much better results in comparison to Abs spectroscopy. Specifically, the cross‐validation parameters were 0.452–0.800 for R^2^Y and 0.392–0.55 for Q^2^Y. Nevertheless, such a performance cannot be considered satisfactory, highlighting that the dilution factor of 10 was rather high to achieve successful vintage discrimination in both cases. Considering the above, further discussion will be dedicated to the chemometric analysis of the non‐diluted and two‐times diluted samples, which achieved the highest Q^2^Y values (Table 4).

**Table 4:** Effect of spectral data pre‐processing methods on the obtained cross‐validation parameters in the case of FL spectroscopy (*n* = 30)

Applied pre‐processing ID	No dilution		Two‐times dilution		Five‐times dilution		Ten‐times dilution	
	R^2^Y	Q^2^Y	R^2^Y	Q^2^Y	R^2^Y	Q^2^Y	R^2^Y	Q^2^Y
1	0.804	0.586	0.497	0.43	0.529	0.44	0.516	0.426
2	0.795	0.602	0.573	0.505	0.61	0.554	0.489	0.452
3	0.742	0.61	0.587	0.518	0.63	0.582	0.656	0.533
4	0.196	0.162	0.686	0.575	0.406	0.37	0.493	0.392
5	0.196	0.162	0.723	0.572	0.497	0.368	0.8	0.55
6	0.52	0.264	0.53	0.394	0.518	0.415	0.539	0.407
7	0.597	0.506	0.553	0.492	0.726	0.578	0.452	0.398
8	0.621	0.497	0.619	0.553	0.72	0.573	0.453	0.397
9	0.309	0.284	0.469	0.408	0.476	0.405	0.452	0.398
10	0.691	0.503	0.599	0.547	0.673	0.556	0.516	0.426
11	0.619	0.487	0.62	0.551	0.719	0.558	0.453	0.398
12	0.309	0.285	0.567	0.405	0.476	0.404	0.592	0.392

PCA showed that the time of measuring significantly contributed to the observed variance, the so‐called ‘batch effect’. The batch effect has been reported in food authenticity studies when using both chromatographic^40^ and spectroscopic methods^41^ and it is related to systematic errors (or differences not related to the composition of the tested sample) induced by the instrumentation when the analysis is split over several days.^42^ For example, in the case of spectroscopy, this may be related to intensity inconsistencies of the optical source illuminating the tested samples with electromagnetic radiation. In our study, this was clearly obvious for all four classes in the relevant PCA score plots for both the non‐diluted and two‐times diluted samples (Fig. S4). The loading plots for all PCA models can be found in the supporting information (Fig. S5). Importantly, such batch effects were not that profound when Abs was used as the analytical signal indicating that FL quenching related to wine compounds may be responsible for such a result. FL quenching occurs when a fluorescent compound (the fluorophore) experiences a reduction in its fluorescence intensity due to the presence of another compound (the quencher) that interacts with it. This can be quite a profound phenomenon when monitoring wine due to the diverse and abundant wine composition in phenolics and tannins. To face this problem, using multiple excitation wavelengths would have been necessary.^34^ However, as already discussed in section ‘FL spectra’, a single *λ*_ex_ (at 250 nm) was used in our study as multiple spectroscopic methods were applied and the experimental and data‐processing load was already high.

**Figure 4:**
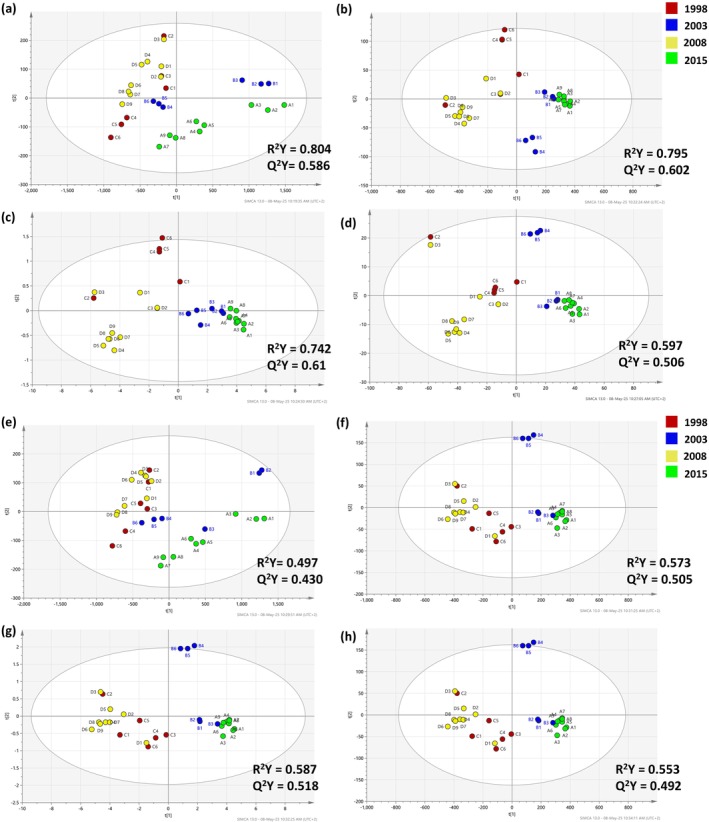
PLS‐DA score plots after applying different pre‐processing methods for the non‐diluted (a–d) and two‐times diluted (e–h) samples. The data were obtained by monitoring FL in the UV–visible region. The loading plots for the respective PLS‐DA models can be found in the supporting information (Fig. S6). (a) and (e): raw; (b) and (f): MSC; (c) and (g): SNV; (d) and (h): MSC followed by SG 1st derivation (2nd polynomial).

The batch effect was expressed the most when no spectral pre‐processing was applied (Fig. S4(a),(e)) highlighting that spectral pre‐processing can compensate such inconsistencies. It is evident that samples belonging to the first batch (IDs 1–3) were clustered together and distant from the other two batches (IDs 4–6 and 7–9) (Fig. S4(a),(e)). For example, all pre‐processing methods eliminated the batch effect for two vintages, namely wines produced in 2008 and 2015. Proceeding to the supervised chemometric analysis, in the case of non‐diluted samples, only four models achieved Q^2^Y > 0.5 representing cases providing acceptable model predictability. For these models (Fig. 4(a)–(d)), R^2^Y fluctuated in the range 0.597–0.804 and Q^2^Y in the range 0.506–0.602, which was significantly better than the respective cases with two‐times dilution (Fig. 4(e)–(h)). Interestingly, PLS‐DA eliminated the batch effect for the samples produced in 2003 (besides 2008 and 2015), when SNV was applied (Fig. 4(c)), achieving the best cross‐validation parameters. Following the same workflow as in the case of Abs, spectral feature selection was performed for the models with the highest proximity between R^2^Y and Q^2^Y. SNV was the spectral pre‐processing achieving the best discriminatory performance for both the non‐diluted and two‐times diluted samples. In both cases, the spectral features exceeding the set cut‐off level (VIP > 1.0) originated mostly from the UV region (Table 3). Interestingly, the highest VIP scores were achieved for the features around 290 nm, a spectral region that produced high VIP scores also for the Abs models. As described earlier, such signals can be attributed to flavanols, compounds with multiple rings inducing fluorescent signals.^43^ Worth mentioning is that the region around 290 nm was amongst those with the most significant excitation in another study focusing on white wine varietal discrimination.^44^

### ATR‐FTIR spectra

**Figure 5:**
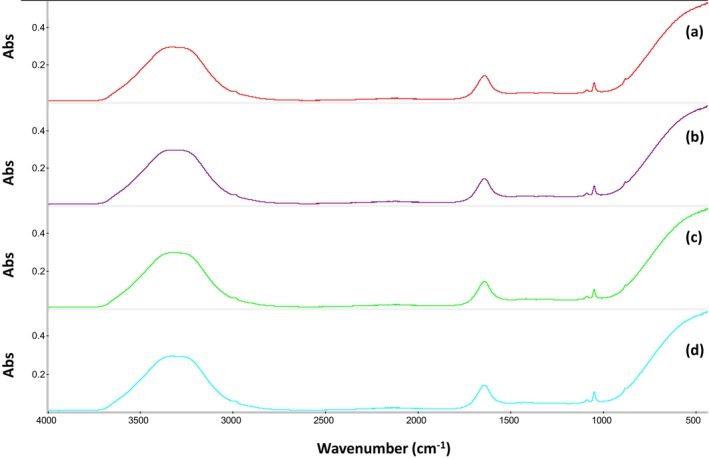
Indicative FTIR spectra for each analyzed vintage. (a): 1998 vintage; (b): 2003 vintage; (c): 2008 vintage; (d): 2015 vintage.

The recorded FTIR spectra were similar for all tested vintages and only differences in the recorded band intensities were monitored (Fig. 5). It was possible to identify six spectral regions exploiting the compositional information provided by MIR spectroscopy. Focusing on the functional group spectral region (4000–1500 cm^−1^), the bands at 3700–3000 and 1700–1600 cm^−1^ are related to the O—H stretching of water.^45^ These two peaks recorded the highest band signal as wine is an aqueous solution. Nevertheless, such signals cannot be considered reliable to discriminate among different classes and for this reason these wavenumbers were not used in the building of chemometric models. In terms of the smaller band at around 3000–2900 cm^−1^, this was attributed to different wine components,^19^ for example, free phenolic acids, C—H stretching of hydrocarbons or O—H stretching of carboxylic acids amongst others. Another small spectral band was noticed between 2300 and 2100 cm^−1^ and it was related to ethanol and sugar C—H combination vibrations and overtones.^19^ Focusing on the fingerprint spectral range (1500–400 cm^−1^), the region between 1550 and 950 cm^−1^ has been identified as of utmost importance for red wine varietal authenticity as it contains the most variance resulting in successful class‐based discrimination.^46^ The bond vibrations in this region were related to phenolic compounds, carboxylic acids, alcohols and other typical wine compounds that depend on wine origin. Lastly, the high signal observed below 800 cm^−1^, also known as the ‘true fingerprint region’, is considered unique for each tested sample and bond assignment was not possible in this case.^47^

### Investigating FTIR spectral data pre‐processing and impact on multivariate analysis

**Figure 6:**
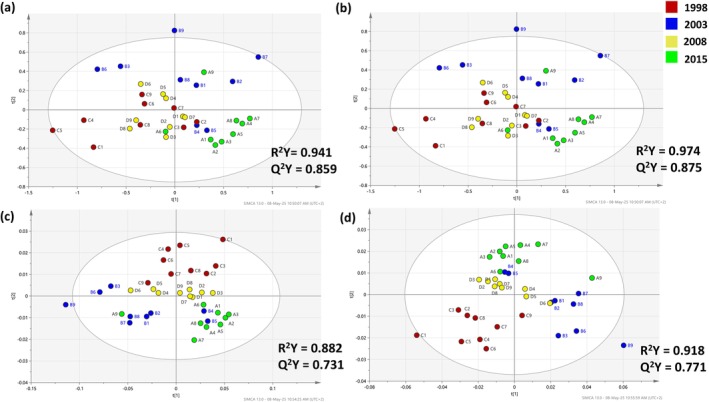
PLS‐DA score plots after applying different pre‐processing methods for the non‐diluted wine samples. The data were obtained by monitoring Abs in the MIR region. The loading plots for the respective PLS‐DA models can be found in the supporting information (Fig. S9). (a): raw; (b): MSC; (c): SG 1st derivation (2nd polynomial); (d): MSC followed by SG 1st derivation (2nd polynomial).

In contrast to UV–visible spectroscopy, diluting the samples would not have been helpful in the ATR‐FTIR analysis, as MIR radiation carries less energy than UV–visible radiation (based on the Planck equation: *E* = *hf*, where *E* is energy, *h* is Planck's constant, *f* is frequency). So, no dilution was performed. Focusing on the spectral pre‐processing, the model accuracy was improved, in terms of R^2^Y (R^2^Y > 0.9 in some cases), when using noise correction or combined methods (Table 5). Nevertheless, the model predictability was rather poor (Q^2^Y < 0.5) in all cases, when all the attained spectral features were used (except the water bands). To overcome this challenge, feature selection was performed based on wine compositional information. The region between 1550 and 950 cm^−1^ was selected resulting in very good model performances in the range 0.836–0.974 for R^2^Y and 0.525–0.875 for Q^2^Y. Focusing on multivariate analysis for four indicative models, class clustering based on the vintage was noticed using PCA (Figs S7, S8) whilst the PLS‐DA models achieved very good performance (Fig. 6). Specifically, MSC was proved to be the most efficient pre‐processing method facing scattering induced by tartrate crystals, such as potassium hydrogen tartrate.^48^ In addition, no batch effect was observed for the MIR measurements. On the downside, in all cases, one sample originating from 2015 (sample A9, Fig. 6) was clustered with the 2003 samples. This was not noticed in the UV–visible fingerprints highlighting the potential differences that can be noticed when analyzing the same samples by different spectroscopic principles.

**Table 5:** Effect of spectral data pre‐processing methods on the obtained cross‐validation parameters in the case of FTIR spectroscopy (*n* = 36)

Applied pre‐processing ID	No dilution, all features*		No dilutionm 1550–950 cm^−1^	
	R^2^Y	Q^2^Y	R^2^Y	Q^2^Y
1	0.297	0.207	0.941	0.859
2	0.382	0.155	0.974	0.875
3	0.382	0.155	0.974	0.875
4	0.885	0.355	0.882	0.731
5	0.922	0.371	0.882	0.731
6	0.569	0.0125	0.836	0.478
7	0.884	0.357	0.918	0.771
8	0.921	0.377	0.922	0.771
9	0.568	0.0159	0.859	0.755
10	0.884	0.357	0.92	0.776
11	0.921	0.377	0.904	0.737
12	0.568	0.0159	0.852	0.525

### External validation of models with optimum cross‐validation parameters

During the investigation of the spectral pre‐processing effect on chemometrics, the cross‐validation parameters, R^2^Y and Q^2^Y, were used as an indicator of the model performance. However, here, more evaluation metrics were employed to ensure that the optimum models were robust and did not suffer from overfitting. Overall, the recognition ability of the models based on Abs and FTIR was excellent, reaching 100% in all three cases (Table 6). However, the Abs‐based models proved more efficient in correctly classifying test samples (not included in the training set) with a prediction ability >95%. Again, the lowest RMSEE was obtained using Abs spectroscopy (when samples were diluted two times) indicating a very good model fit. In terms of FL, it achieved the worst recognition and prediction abilities whilst the highest RMSEE was also noticed. This is in line with the obtained cross‐validation parameters that were the lowest amongst all five models (see Tables 2, 4 and 5). Importantly, sufficient model reliability was achieved in all cases as always *P* < 0.05 denoting significant models.^49^ Also, based on the performed permutation tests, all models were considered valid as the obtained Q^2^Y values from the permuted data (displayed on the left) were smaller than the actual Q^2^Y value (displayed on the right) (see Fig. S10) and the regression line had a negative intercept value on the *y*‐axis.^50^ In the final stage of this study, six new samples of PDO Xinomavro with 2019 vintage were analyzed. In detail, Abs (two‐times diluted and pre‐processed by SNV) and FTIR (MSC pre‐processing) measurements were performed as these were the models with the best recognition and prediction ability. Non‐supervised PCA was employed to identify trends in the extended dataset. Indeed, the samples with 2019 vintage were clearly clustered in the score plots (Fig. S11) from the rest of the samples, and in the case of FTIR two samples were considered as outliers. Obtaining such a result demonstrates the robustness of the developed four‐class model as non‐supervised chemometric models do not take into account the sample classes. In addition, this result indicates that more vintages could be added and potentially effectively discriminated by the models. However, more samples would be necessary to be confident about that.

**Table 6:** Model evaluation metrics for the models with the optimum cross‐validation parameters

Spectroscopic method	Dilution	Spectral pre‐processing	Recognition ability (%)	Prediction ability (%)	RMSEE	CV‐ANOVA *P* value	Permutation test
Abs	Two times	SNV	100	95	0.08	<0.001	No overfitting was indicated
	Five times	MSC	100	96	0.12	<0.001	
FL	No dilution	SNV	93	67	0.17	0.005	
	Two times		77	65	0.27	<0.001	
FTIR	No dilution	MSC	100	80	0.09	<0.001	

## CONCLUSIONS

Comprehensive spectroscopic fingerprints were acquired using three spectroscopic principles to assess the vintage of PDO Xinomavro Naoussa red wine. In each case, spectral interpretation was presented and the analytically interesting spectral regions were identified. The achieved sample clustering was satisfactory in all cases with Abs in UV–visible and MIR achieving excellent class discrimination. Worth mentioning is the impact of sample dilution in UV–visible spectroscopy, a step that significantly affected the model ability to correctly predict the vintage. Special focus placed on the impact of spectral pre‐processing methods on multivariate analysis, represented by non‐supervised PCA and supervised PLS‐DA. In most cases, the models based on pre‐processed spectral data achieved better cross‐validation parameters highlighting the importance of spectral pre‐processing. In terms of the most efficient pre‐processing method, the models with the best cross‐validation parameters were realized when the scatter correction methods, namely MSC and SNV, were used. Additionally, our study highlighted that by optimizing spectral pre‐processing, one can achieve better class discrimination in food authenticity studies. This optimization step is not laborious, is part of the data handling and can be even automated or programmed, further accelerating the decision process. Nevertheless, further chemometric model validation is needed by adding more vintages or external samples produced during the already tested vintages. All in all, a cost‐efficient, rapid and non‐destructive workflow was presented providing a solution that can be of high interest for the wine industry. Work is underway to fuse spectral data from different sources potentially achieving a better discriminatory power by the respective models.

## CONFLICT OF INTEREST

The authors do not have any conflict of interest.

## Supporting information


**DATA S1** Supporting Information.

## Data Availability

The data that support the findings of this study are available from the corresponding author upon reasonable request.
