# Supplementary material for: The impact of spectral data pre‐processing on the assessment of red wine vintage through spectroscopic methods
Source: J Sci Food Agric. 2025 May 12;105(11):5986–98. doi: 10.1002/jsfa.14351 (PMC12260330; doi:10.1002/jsfa.14351)
Supplement: Supplementary file 1 — DATA S1 Supporting Information. [file JSFA-105-5986-s001.docx]

**SUPPLEMENTARY MATERIALS**

**The impact of spectral data pre-processing on the assessment of red wine vintage year through spectroscopic methods**

**Aristeidis S. Tsagkaris^a*^, Natasa Kalogiouri^b^, Viola Tokarova^c^, Jana Hajslova^a^**

a: Department of Food Analysis and Nutrition, Faculty of Food and Biochemical Technology, University of Chemistry and Technology Prague, Technická 5, 166 28 Prague 6– Dejvice, Prague, Czech Republic

b: Laboratory of Analytical Chemistry, Department of Chemistry, Aristotle University of Thessaloniki, Thessaloniki, Greece

c: Department of Chemical Engineering, Faculty of Chemical Engineering, University of Chemistry and Technology Prague, Technická 5, Prague 6–Dejvice, 166 28 Prague, Czech Republic

contact: [tsagkara@vscht.cz](mailto:tsagkara@vscht.cz)


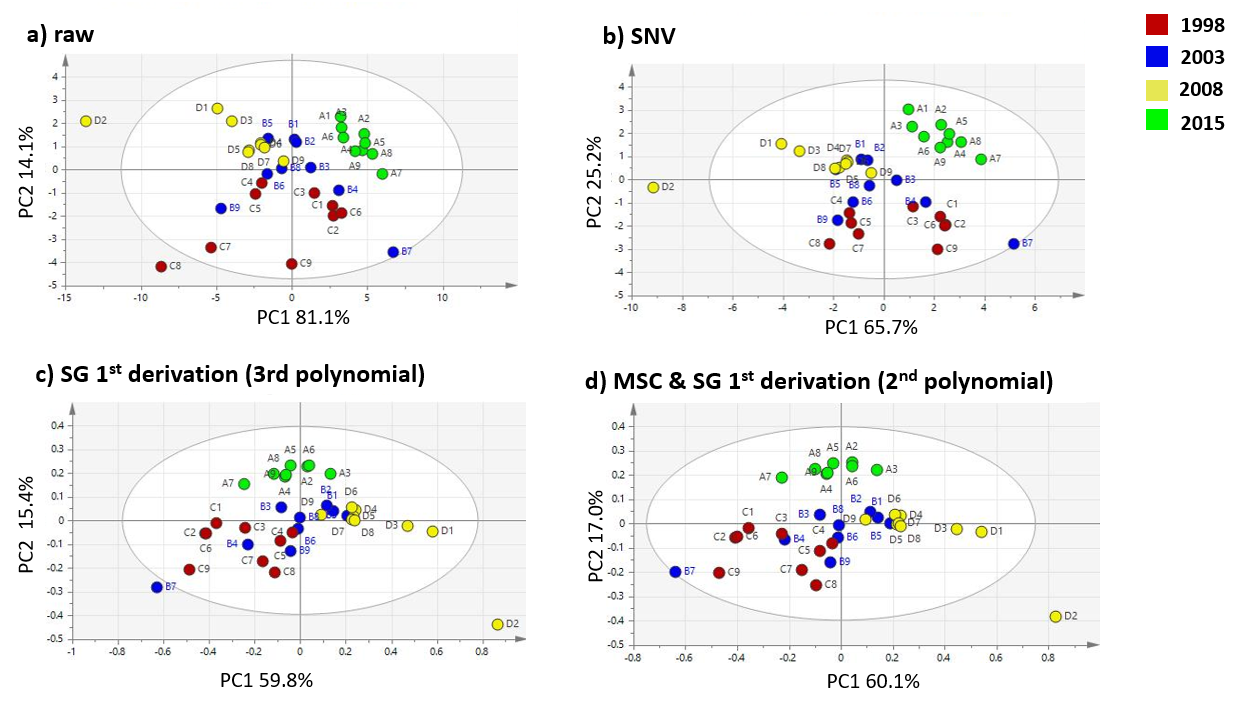


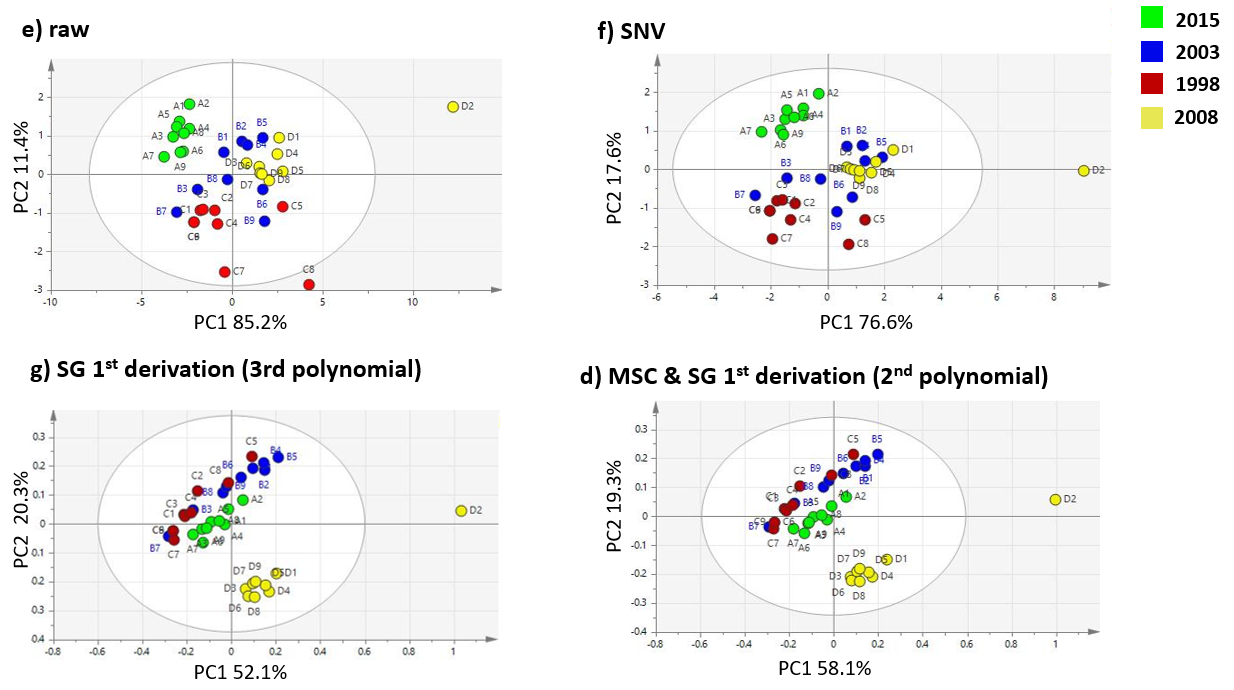


**Fig. S1.** PCA score plots after applying different pre-processing methods for the 2-times (a-d) and 5-times diluted (e-h) samples. The data were obtained by monitoring the Abs at UV-VIS.


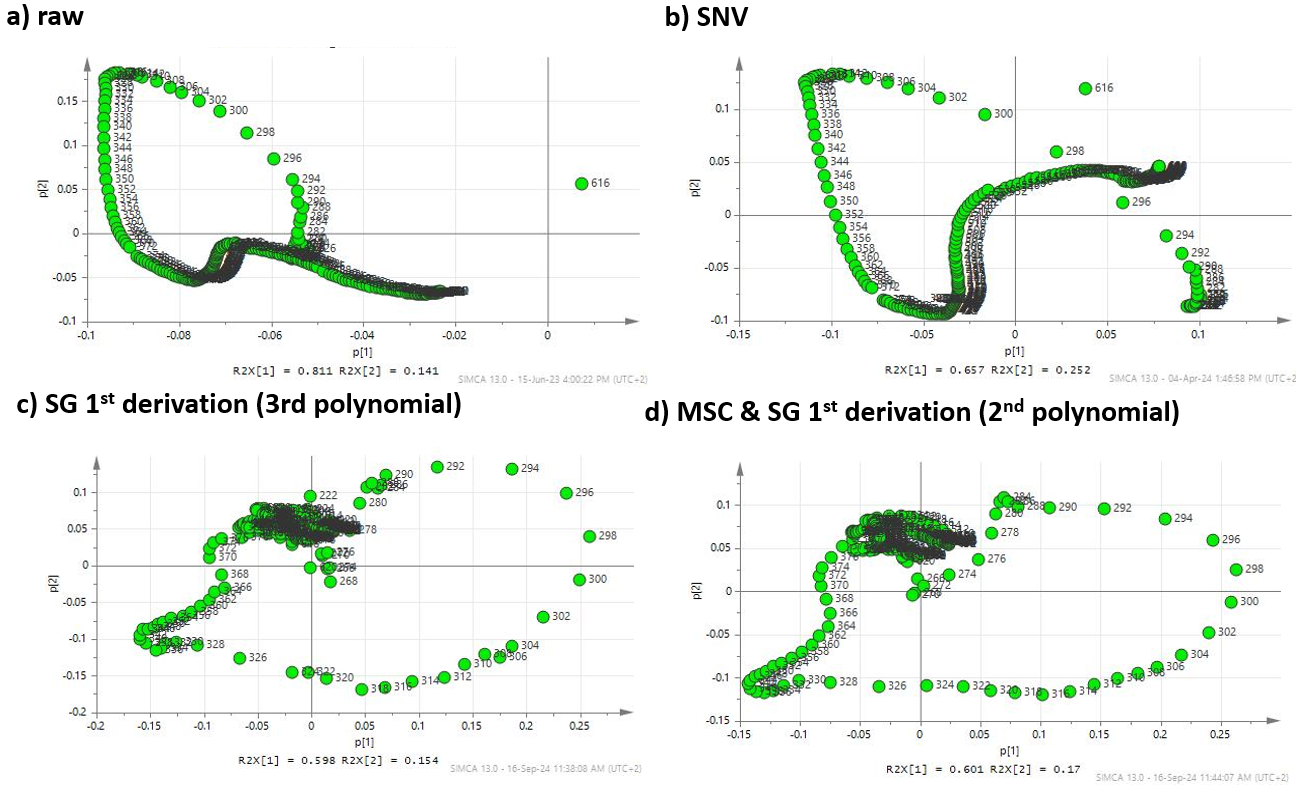


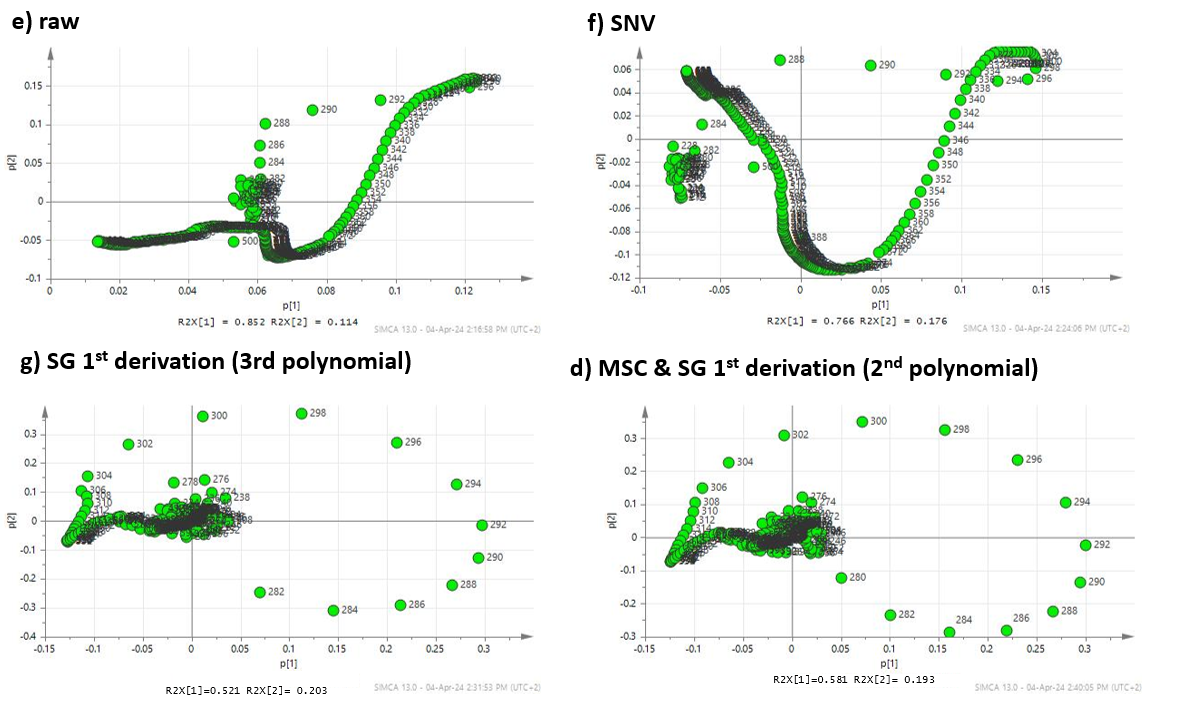


**Fig. S2.** PCA loading plots after applying different pre-processing methods for the 2-times (a-d) and 5-times diluted (e-h) samples. The data were obtained by monitoring the Abs at UV-VIS.


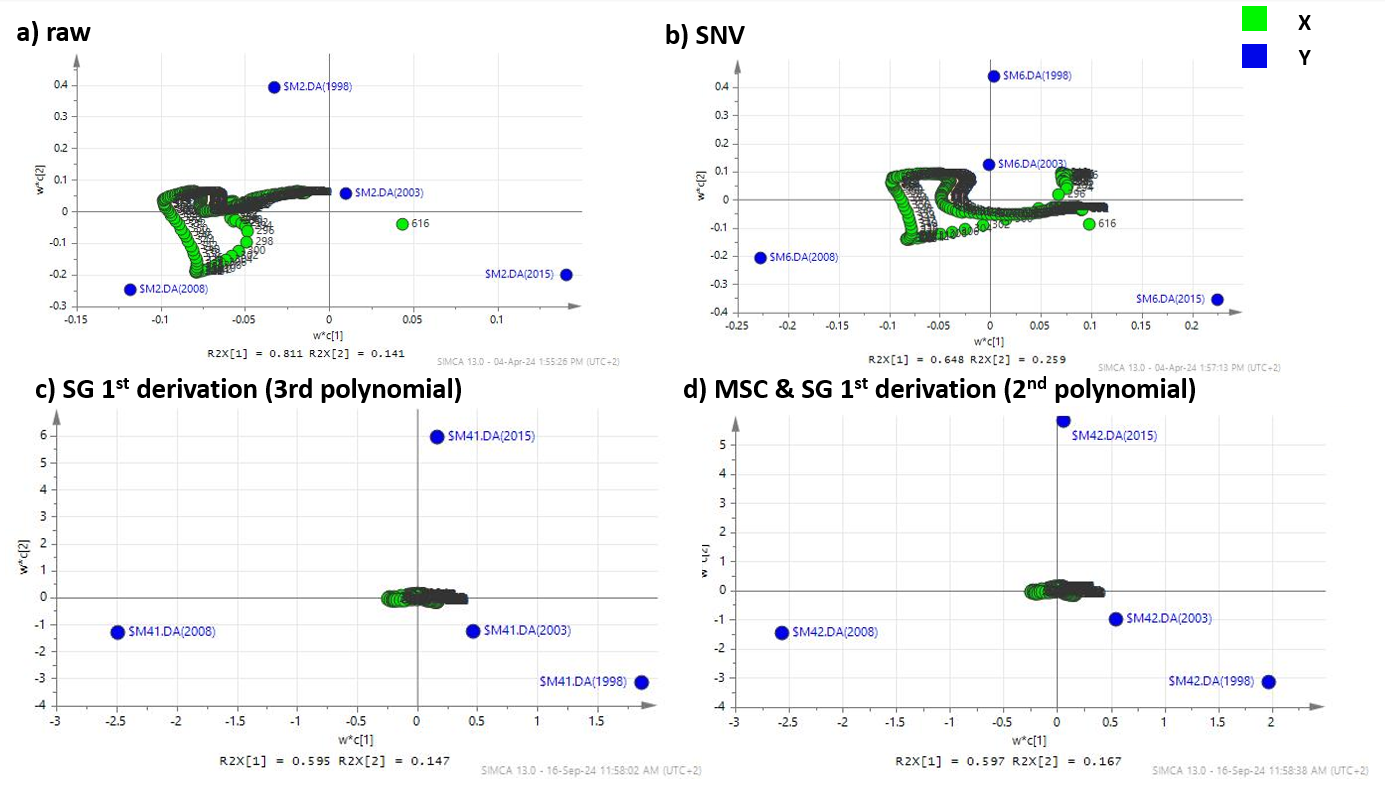


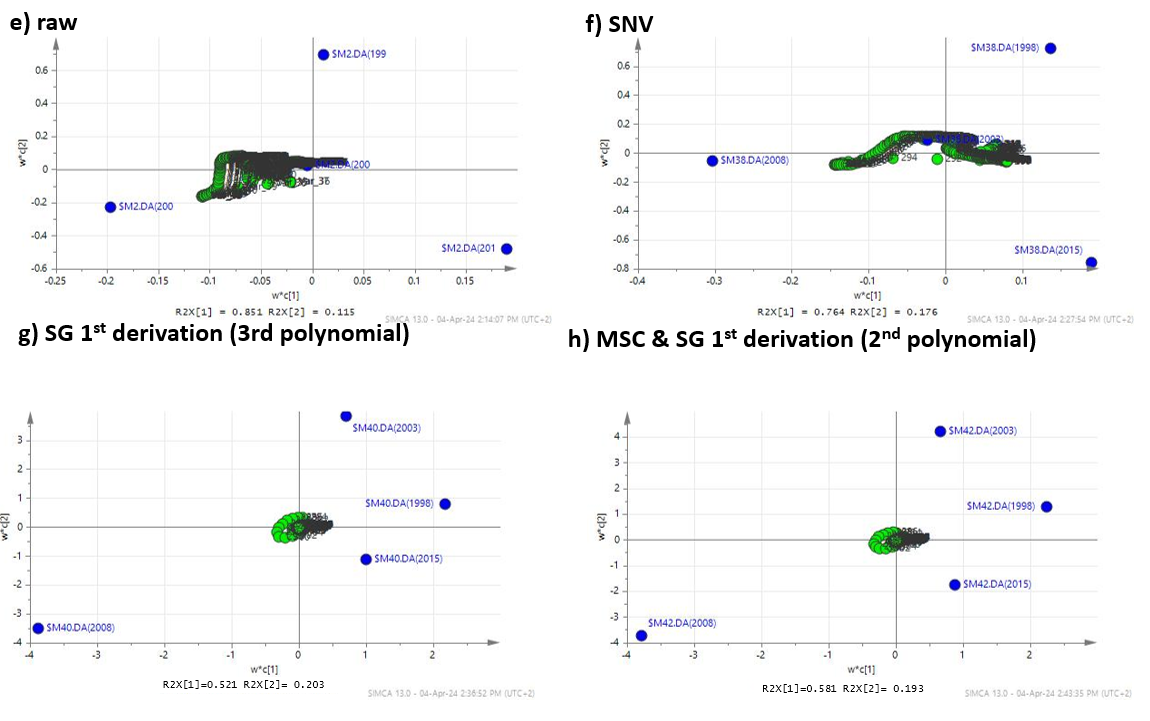


**Fig. S3.** PLS-DA loading plots after applying different pre-processing methods for the 2-times (a-d) and 5-times diluted (e-h) samples. The data were obtained by monitoring the Abs at UV-VIS.


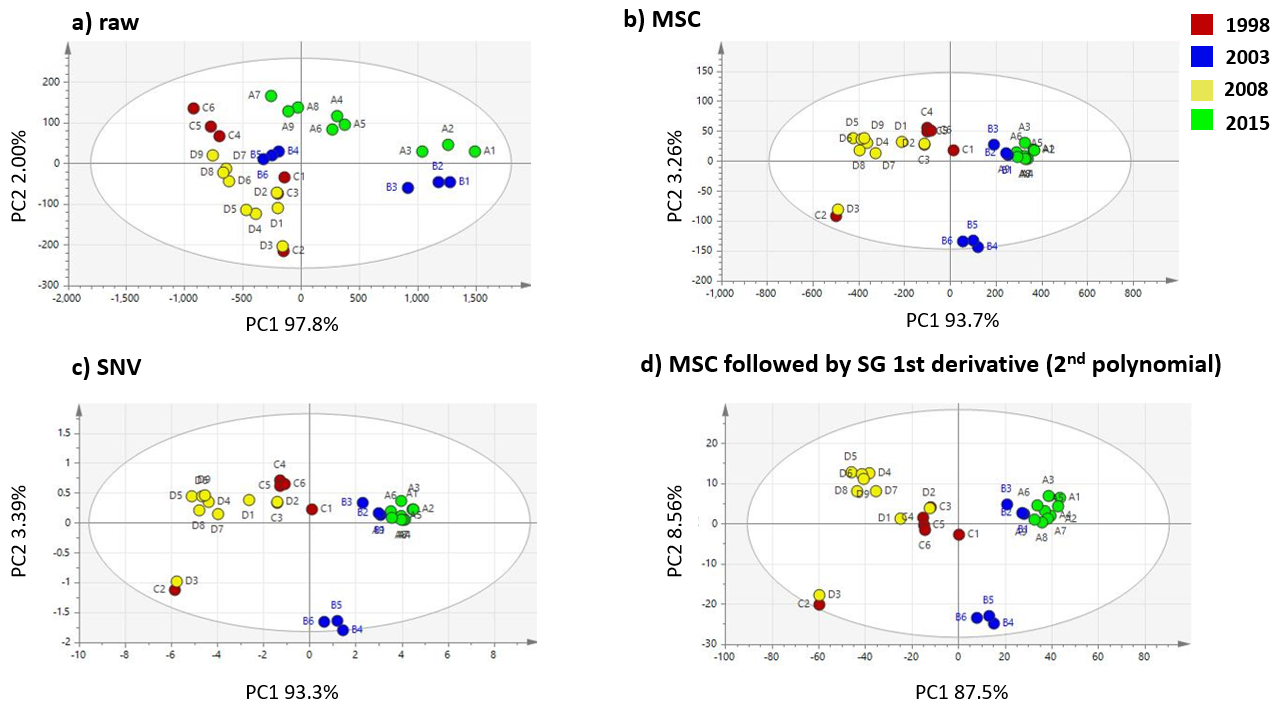


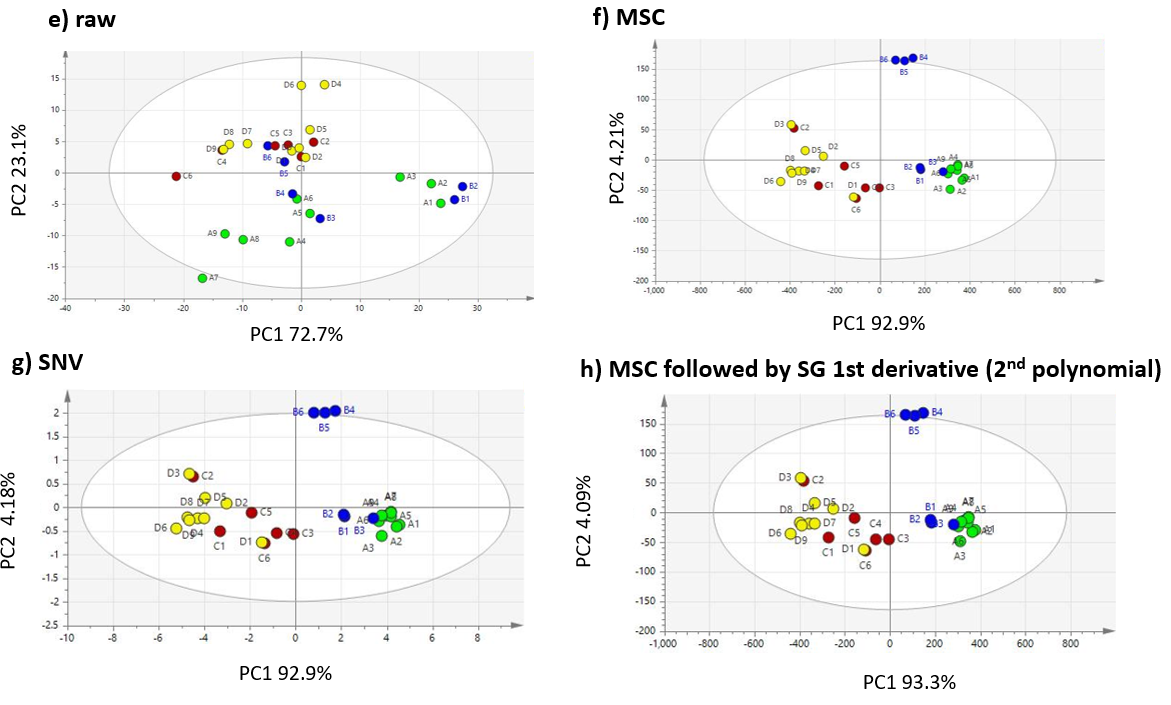


**Fig. S4.** PCA score plots after applying different pre-processing methods for the non-diluted (a-d) and 2-times diluted (e-h) samples. The data were obtained by monitoring the FL at UV-VIS.


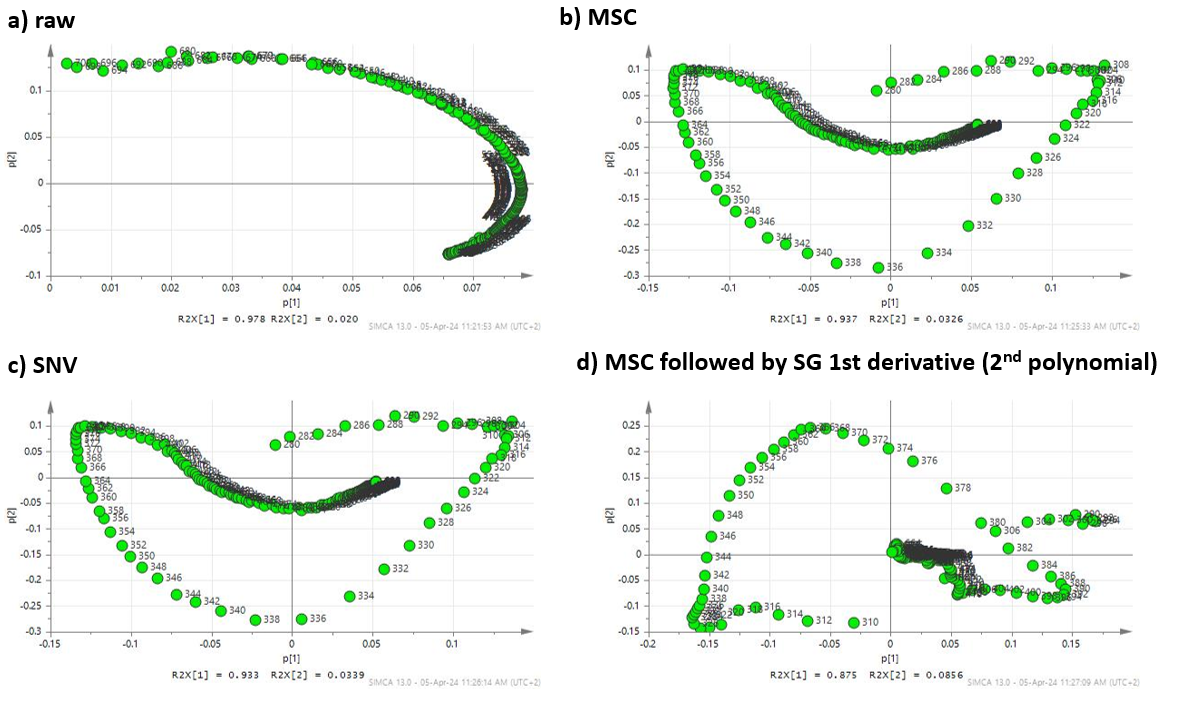


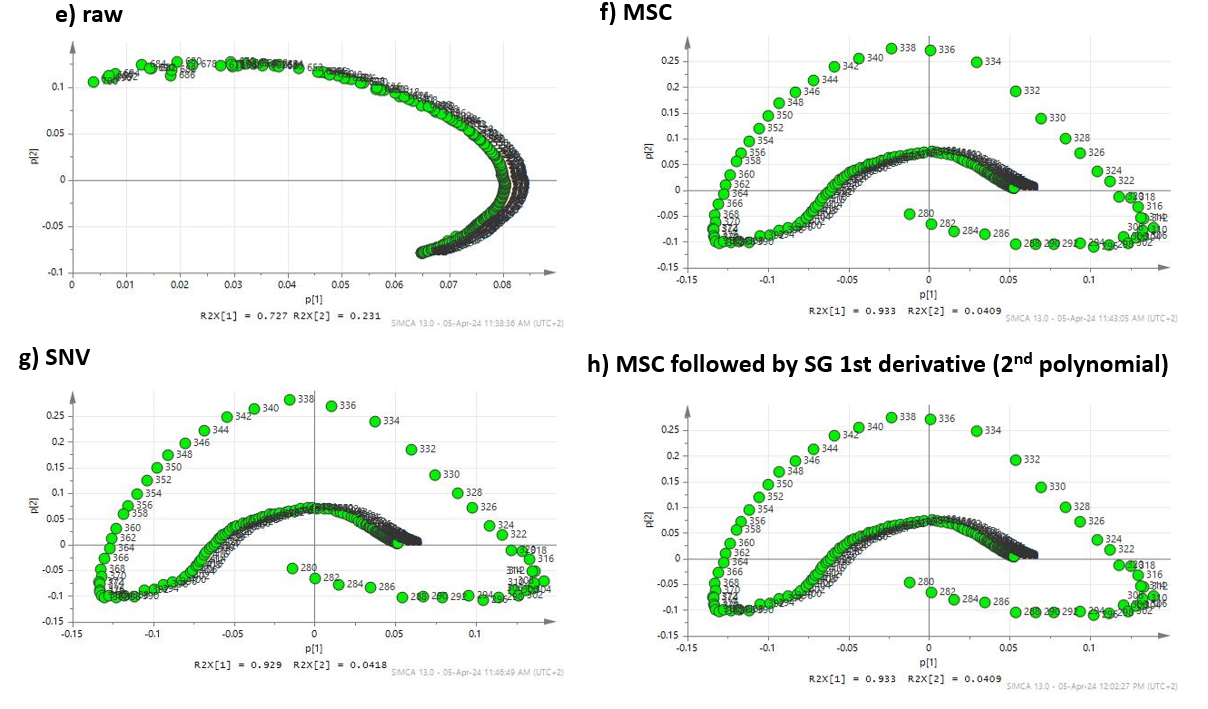


**Fig. S5.** PCA loading plots after applying different pre-processing methods for the non-diluted (a-d) and 2-times diluted (e-h) samples. The data were obtained by monitoring the FL at UV-VIS.


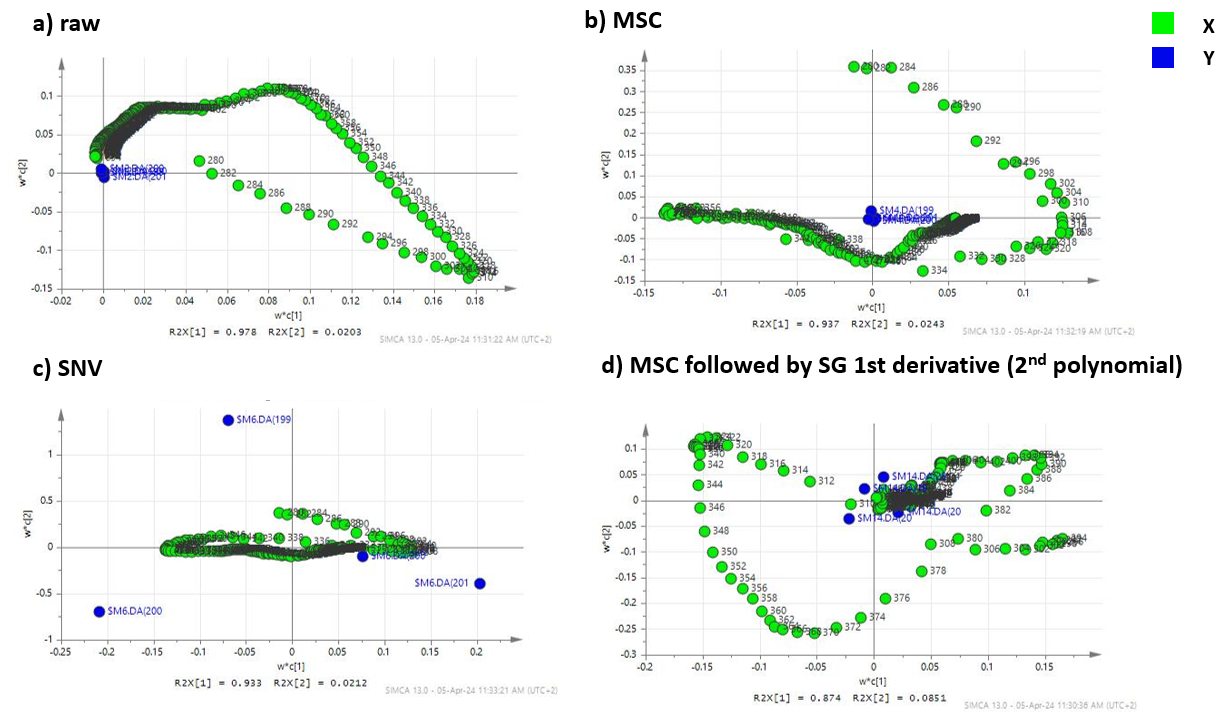


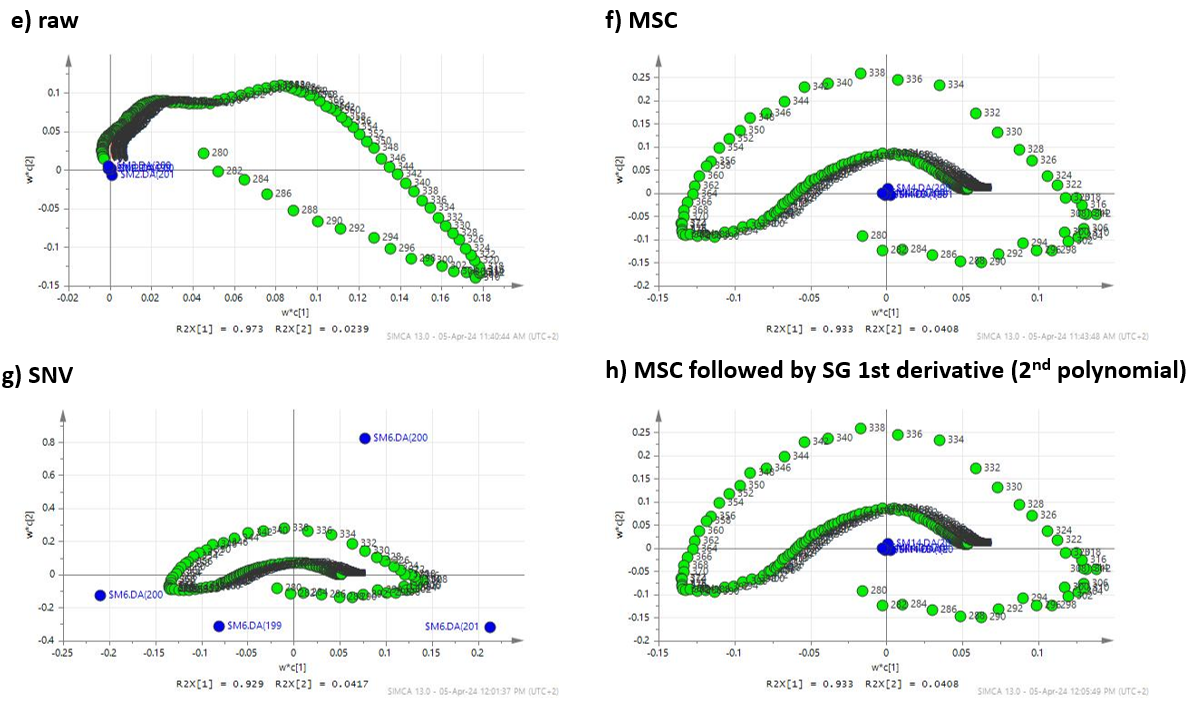


**Fig. S6.** PLS-DA loading plots after applying different pre-processing methods for the non-diluted (a-d) and 2-times diluted (e-h) samples. The data were obtained by monitoring the FL at UV-VIS.


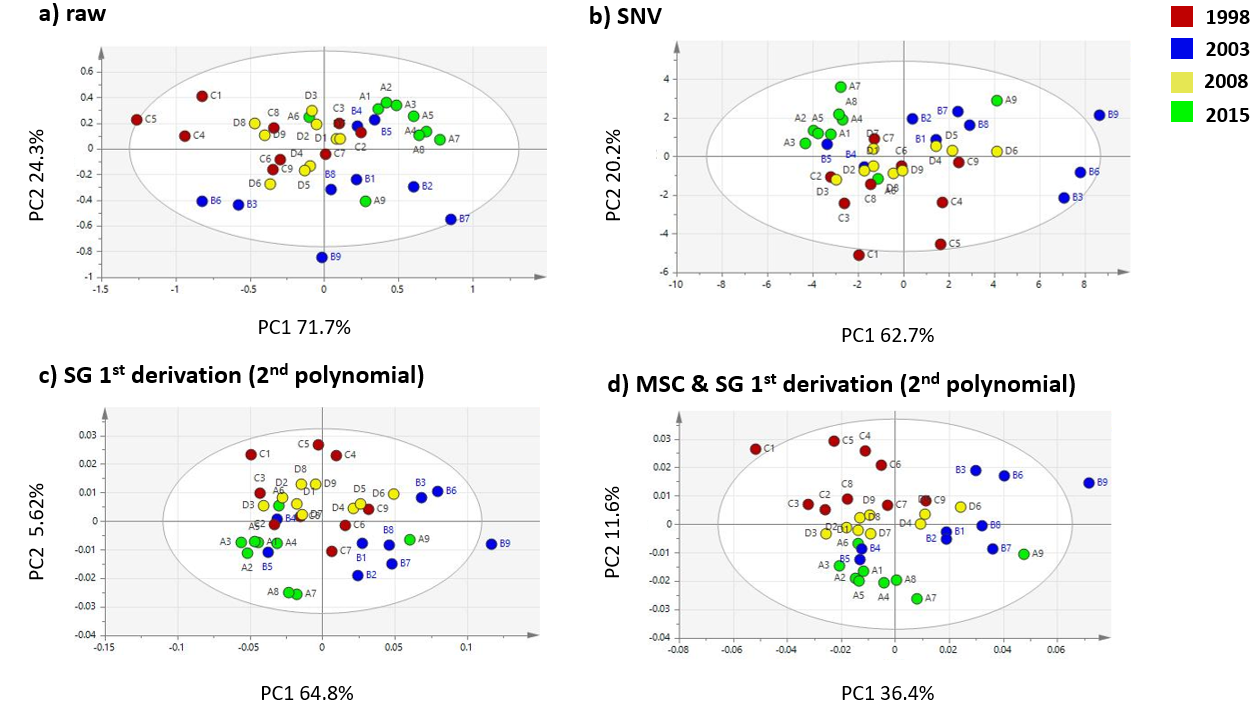


**Figure S7.** PCA score plots after applying different pre-processing methods for the non-diluted wine samples. The data were obtained by monitoring the absorbance at MIR.


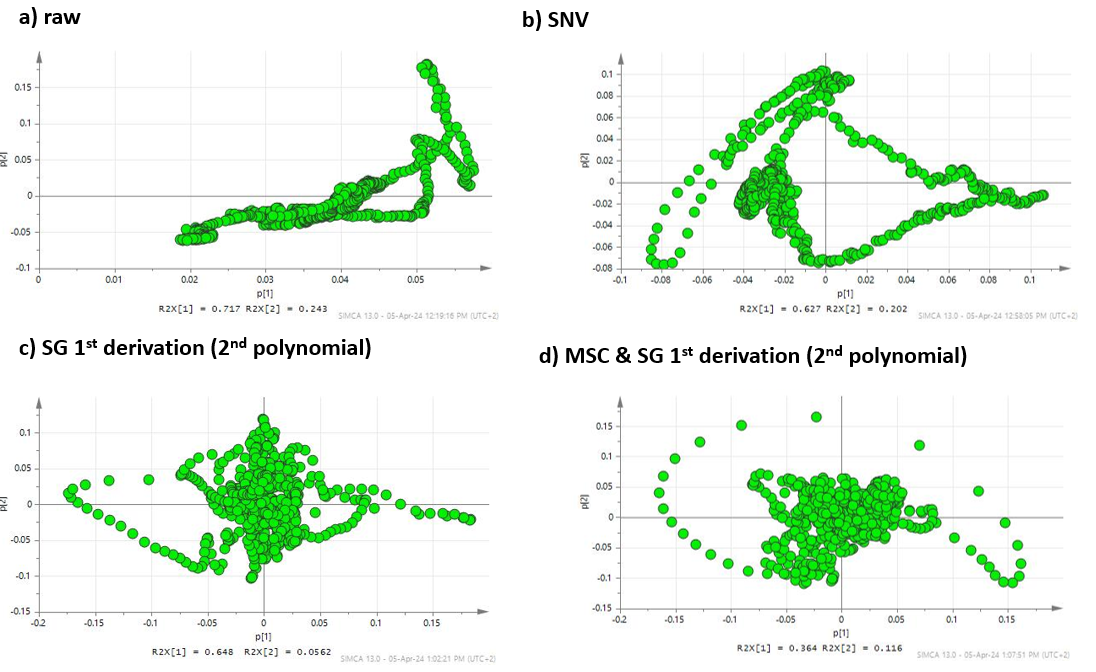


**Figure S8.** PCA loading plots after applying different pre-processing methods for the non-diluted wine samples. The data were obtained by monitoring the absorbance at MIR.


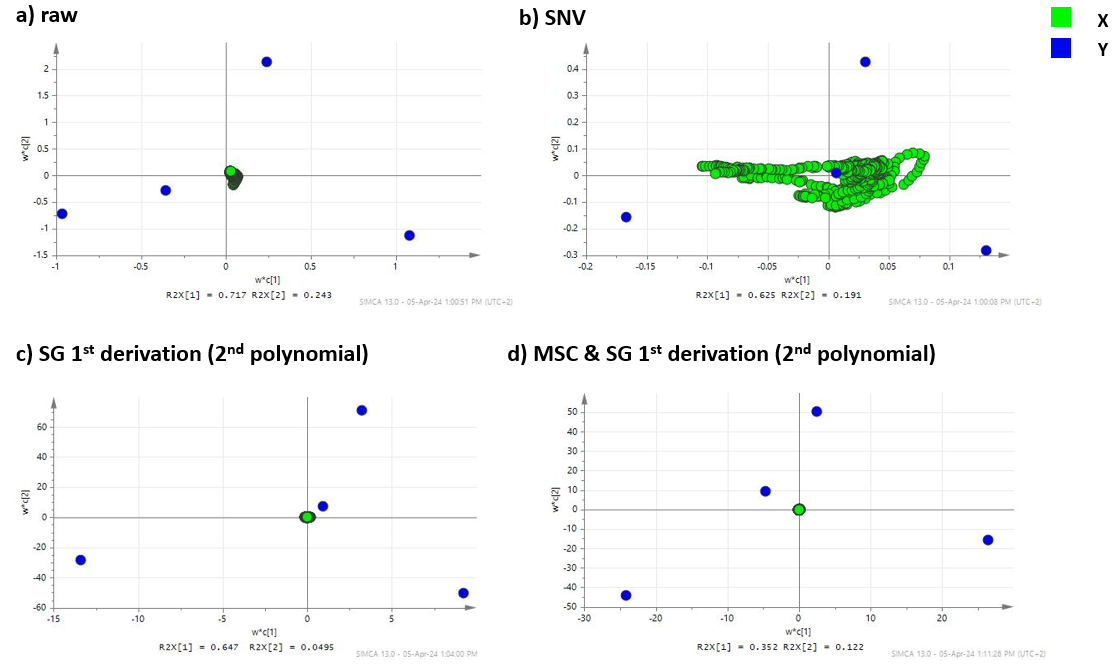


**Fig*.* S9.** PLS-DA loading plots after applying different pre-processing methods for the non-diluted samples. The data were obtained by monitoring the absorbance at MIR.

**Fig*.* S10.** Correlation between permutated and actual cross-validation parameters for each of the 5 selected models.


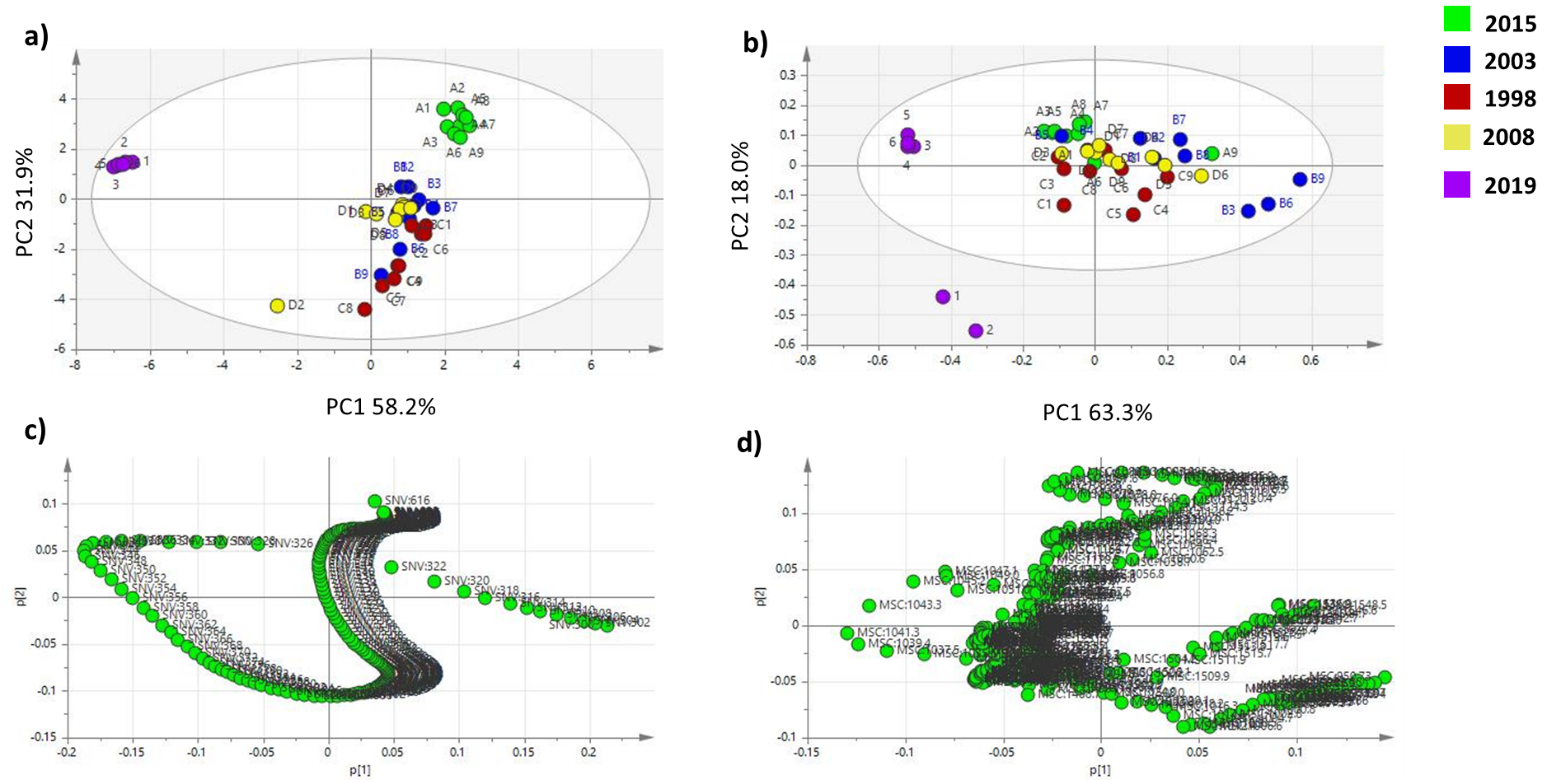


**Fig*.* S11.** PCA score and loading plots for the Abs (Fig. 4a and c) and FTIR (Fig. b and d) measurements including 6 new samples with 2019 vintage.
